# Repair of a Giant Inguinal Hernia

**DOI:** 10.7759/cureus.12327

**Published:** 2020-12-27

**Authors:** Adam Misseldine, Cole Kircher, Saad Shebrain

**Affiliations:** 1 Department of General Surgery, Western Michigan University Homer Stryker M.D. School of Medicine, Kalamazoo, USA

**Keywords:** abdominal compartment syndrome, loss of domain, intra-abdominal pressure, giant inguinal hernia

## Abstract

We report a case of a giant inguinal hernia (GIH) that underwent open surgical repair with mesh. The patient had a massive transcompartmental redistribution of abdominal contents from the abdominopelvic cavity to the hernia sac in the scrotum, with subsequent effects on the mechanical nature of the abdominal wall muscles. Repair of this type of giant hernia is challenging as it can raise the intra-abdominal pressure, therefore increasing the risk of abdominal compartment syndrome (ACS). The large size and chronicity of the hernia, associated with deranged mechanical forces/properties of the abdominal wall, made the management of this complex case unique and interesting. In similar cases of massive incisional or ventral hernias, the term “loss of domain” (LOD) is used. In such types of massive hernias, it is important to carefully plan and monitor for adverse physiological effects associated with increased abdominal pressure.

## Introduction

Inguinal hernias are extremely common in the general population. Approximately 800,000 inguinal hernia repairs are performed in the United States annually. Statistically, one in four men and one in 50 women will acquire an inguinal hernia throughout their lifetime, making this a very prevalent issue [1]. Inguinal hernias (i.e., protrusions of abdominal contents into the inguinal canal) are caused by a weakening in the tissues of the abdominal wall. They are classified as direct or indirect, depending on whether the herniation occurs via the internal or external inguinal ring, respectively. Furthermore, inguinal hernias may be classified as giant inguinal hernias (GIH) if they fall below the midpoint of the thigh in the standing position, as is seen in this case [2]. Risk factors associated with inguinal hernia include family history, chronic obstructive pulmonary disease (COPD), connective tissue disorders, and conditions that may raise the intra-abdominal pressure, such as obesity, cough, and strenuous lifting [1, 3].

## Case presentation

**Figure 1:**
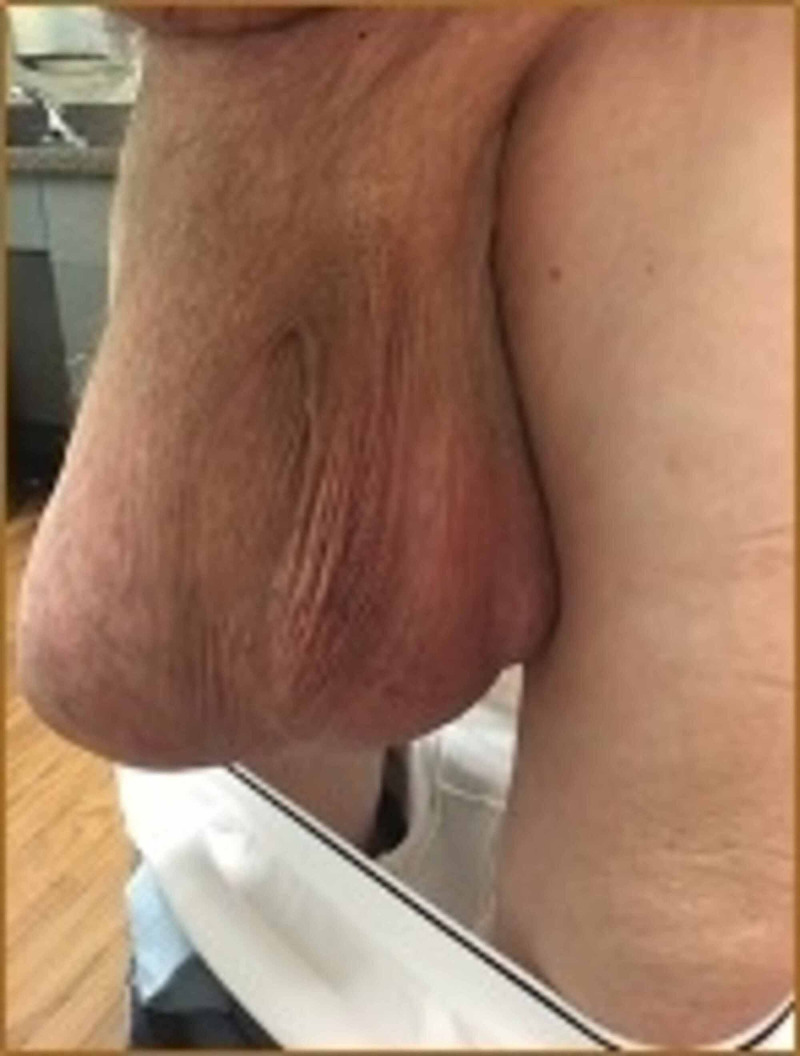
Physical exam (standing) demonstrating a giant inguinoscrotal hernia

**Figure 2:**
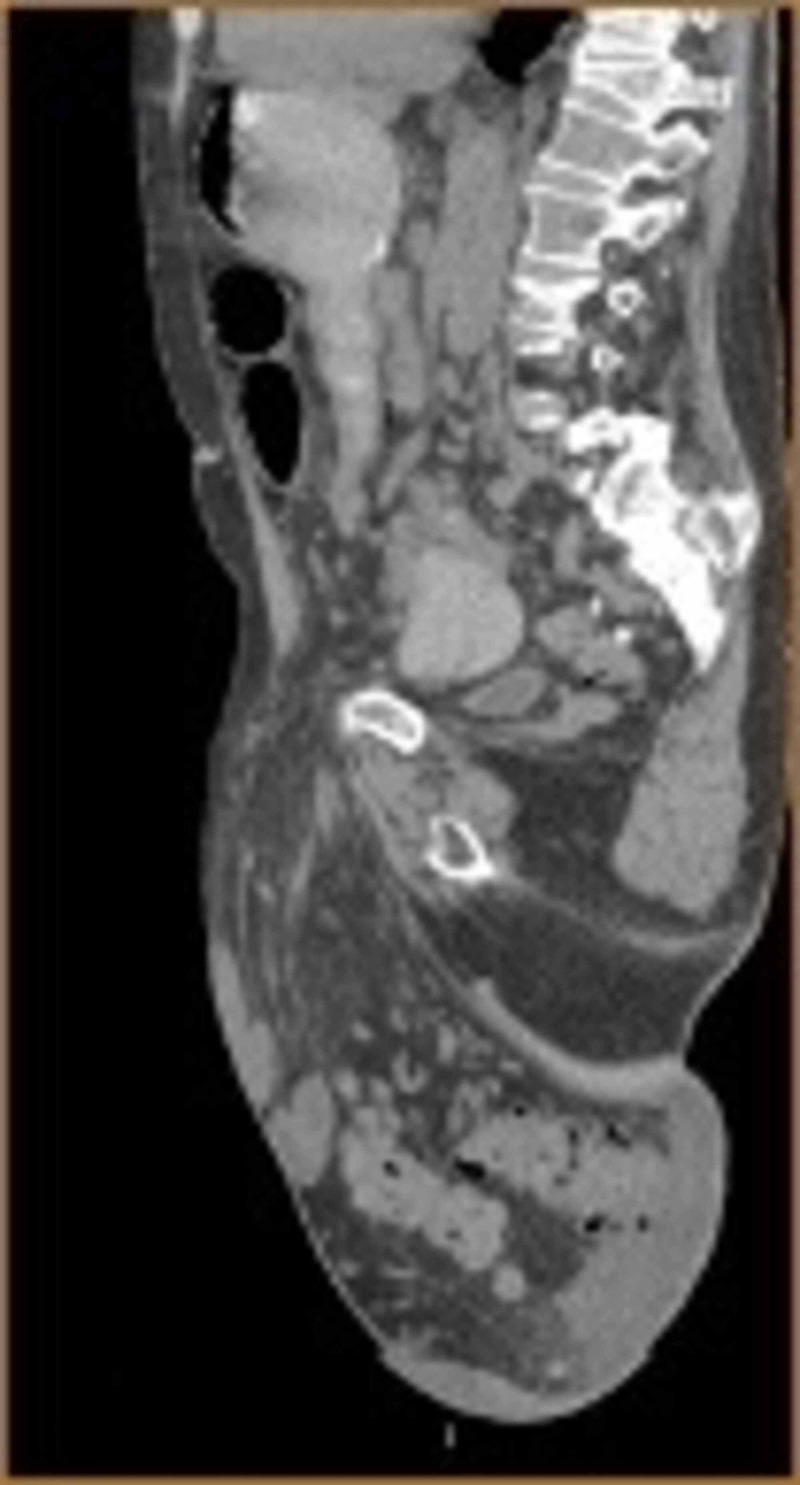
CT scan of the abdomen and pelvis (sagittal view) demonstrating massive scrotal contents

A 76-year-old male with a history of mitral regurgitation, endocarditis, and a GIH was seen in the emergency department for symptoms of small bowel obstruction (SBO). On examination, he had a scaphoid abdomen with a giant, non-reducible right inguinoscrotal hernia, extending down to the level of his knee (Figure 1). Computerized tomography (CT) of the abdomen and pelvis was performed, revealing a partial small bowel obstruction and a large inguinal hernia that appeared to contain the majority of the small bowel and colon (Figure 2). According to the patient, the hernia had been progressively increasing in size over the past 20 years, adversely affecting his quality of life and leading to a loss of normal micturition (due to distortion of the urethra, requiring self-catheterization), back pain, postural change, and difficulty ambulating.

The patient was admitted for a partial small bowel obstruction and a urinary tract infection, both of which were treated non-operatively. The patient was scheduled for an elective hernia repair with preoperative medical and cardiac optimization a few weeks later.

**Figure 3:**
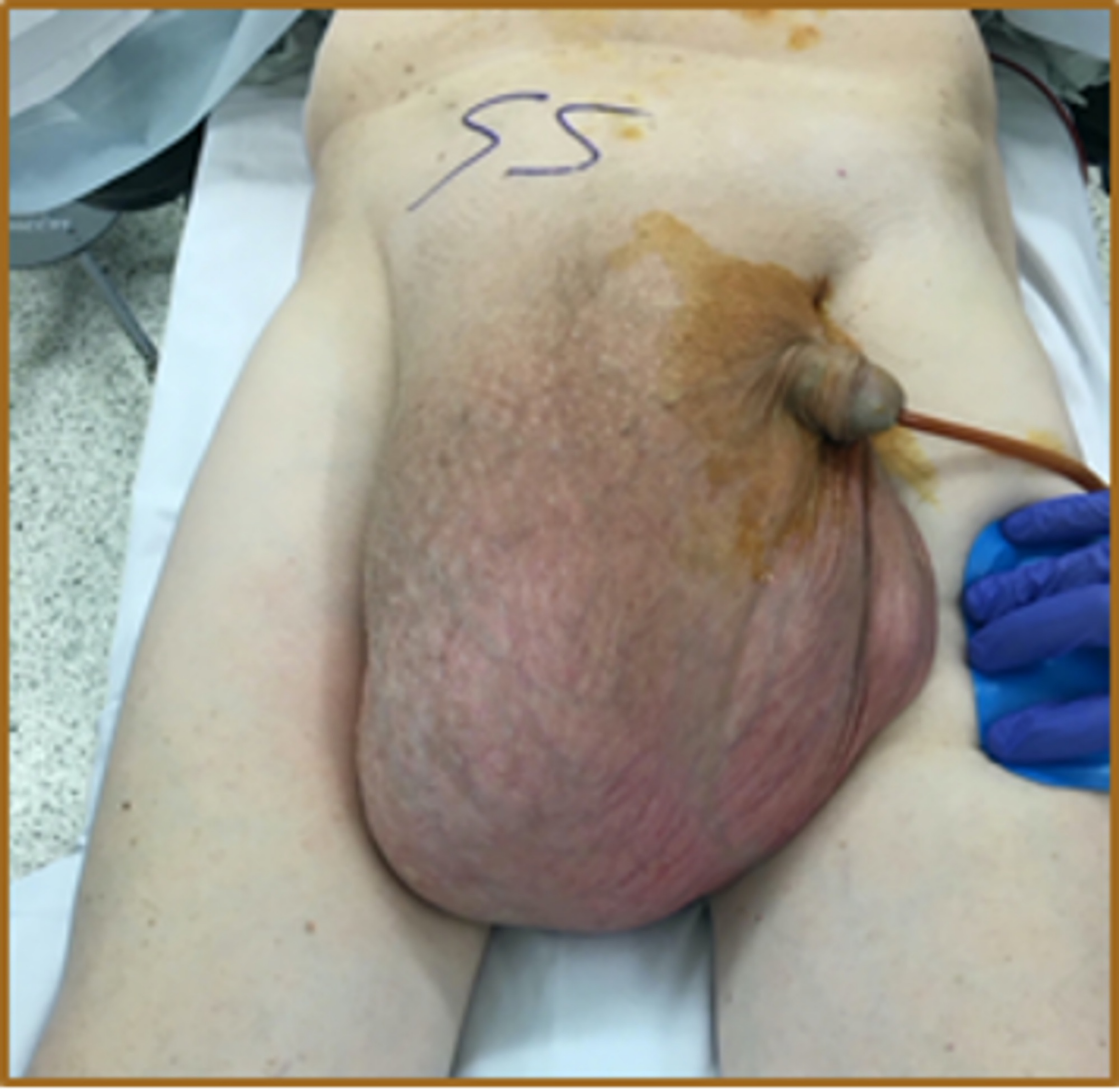
Foley catheter in place, prior to skin incision

**Figure 4:**
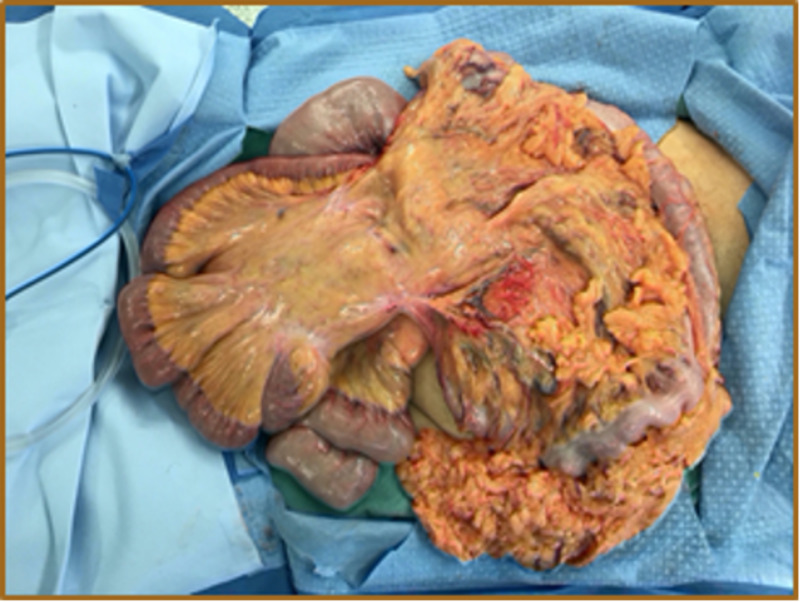
Hernia contents including most of the small bowel, colon, and omentum

**Figure 5:**
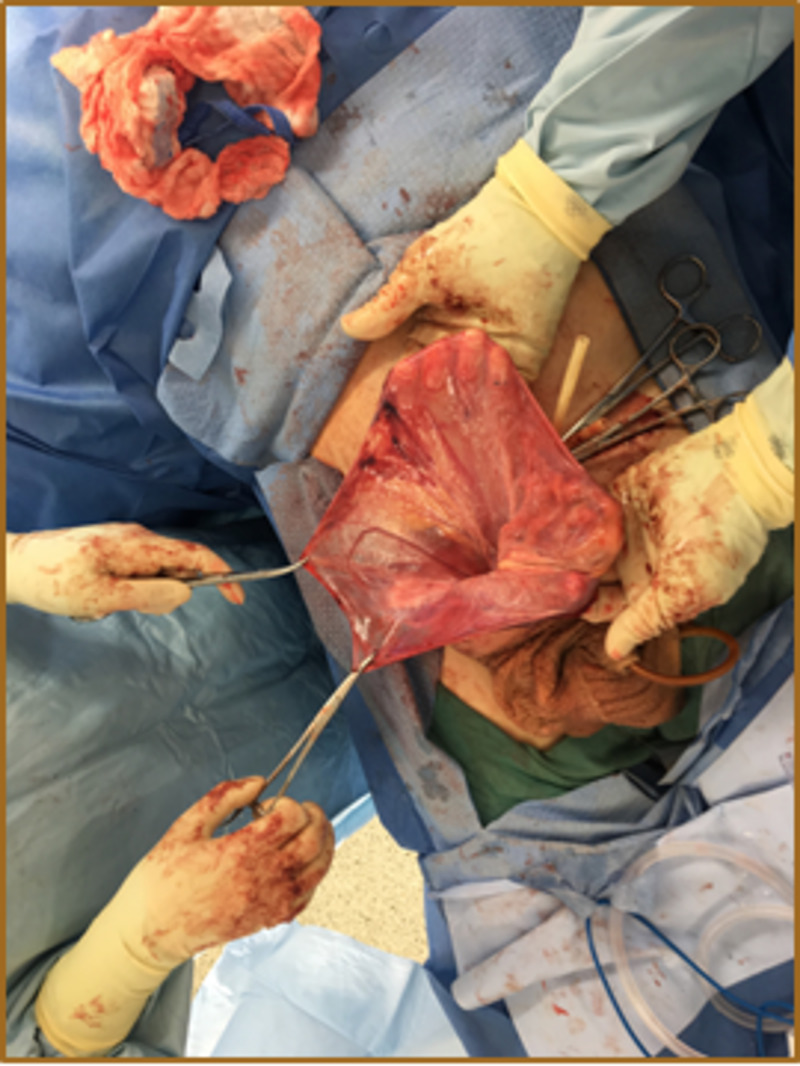
Large hernia sac prepared for excision and high ligation, after reduction of hernia contents

**Figure 6:**
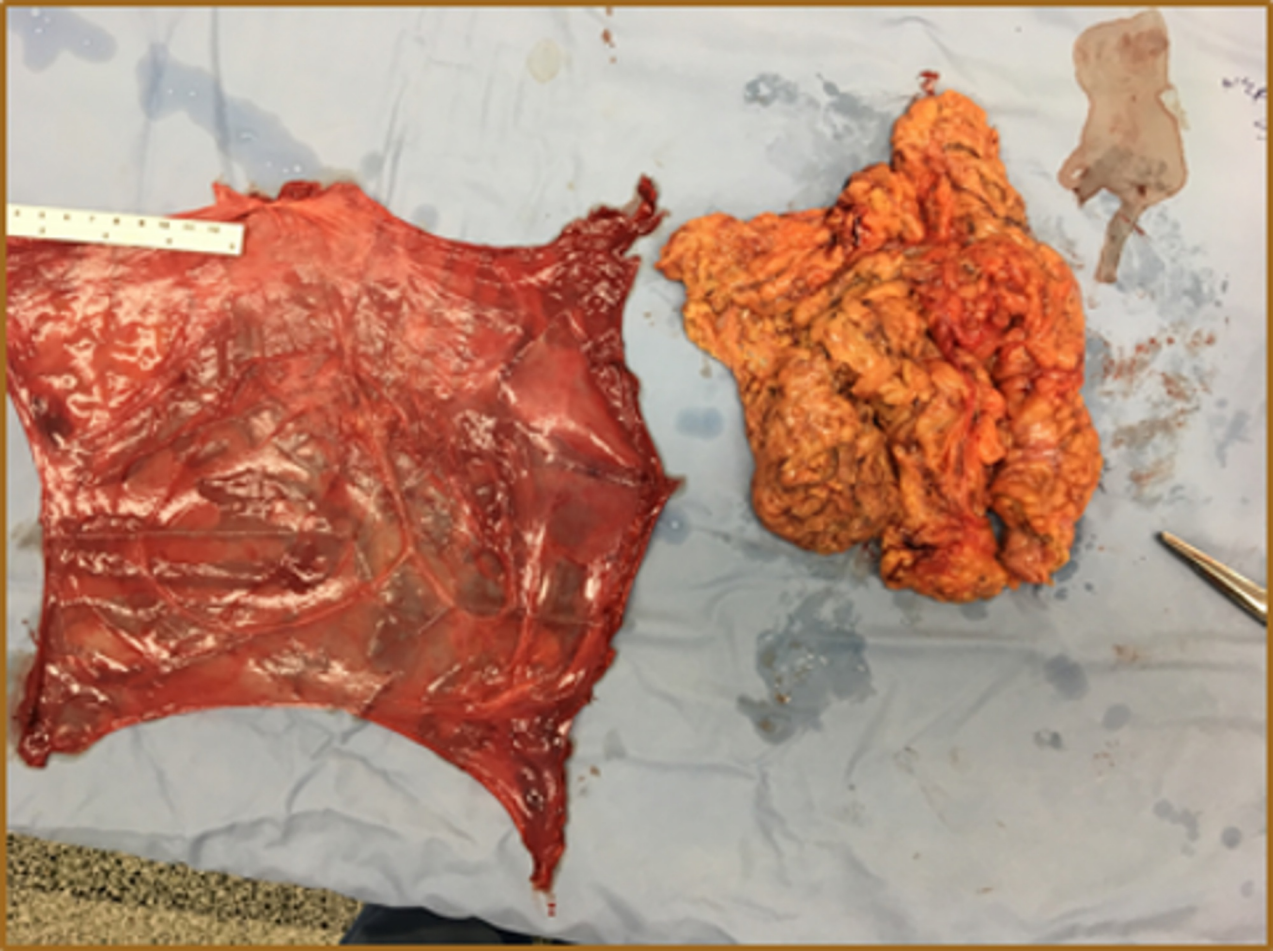
Gross picture of excised hernia sac and omentum

Preoperatively, the patient was placed on a gentle bowel preparation for the possibility of bowel resection. On the day of surgery, he received venous thromboembolism prophylaxis and antibiotics. Under general anesthesia, a transverse right inguinal incision was made. It was noticed that the external oblique aponeurosis was atrophic, overstretched, and adherent to the hernia sac. The sac was opened, and the spermatic cord structures were identified, dissected, and protected. The contents within the hernia sac included the majority of the small bowel, appendix, cecum, ascending and transverse colon, and most of the greater omentum (Figures 3, 4). Due to decreased abdominal cavity volume and chronicity of herniation with massive contents, the patient was placed in a Trendelenburg position during the reduction of the herniated scrotal structures. A partial omentectomy was necessary to minimize intra-abdominal pressure (Figures 5, 6).

**Figure 7:**
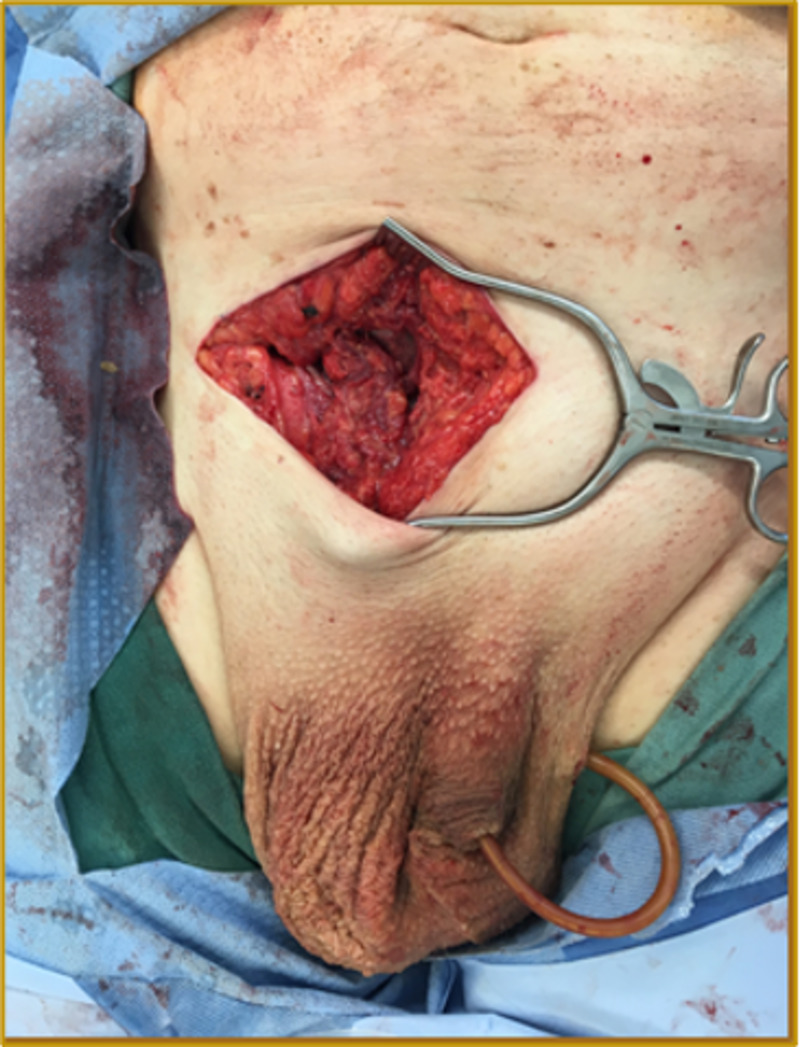
Completed hernia repair with prolene mesh

**Figure 8:**
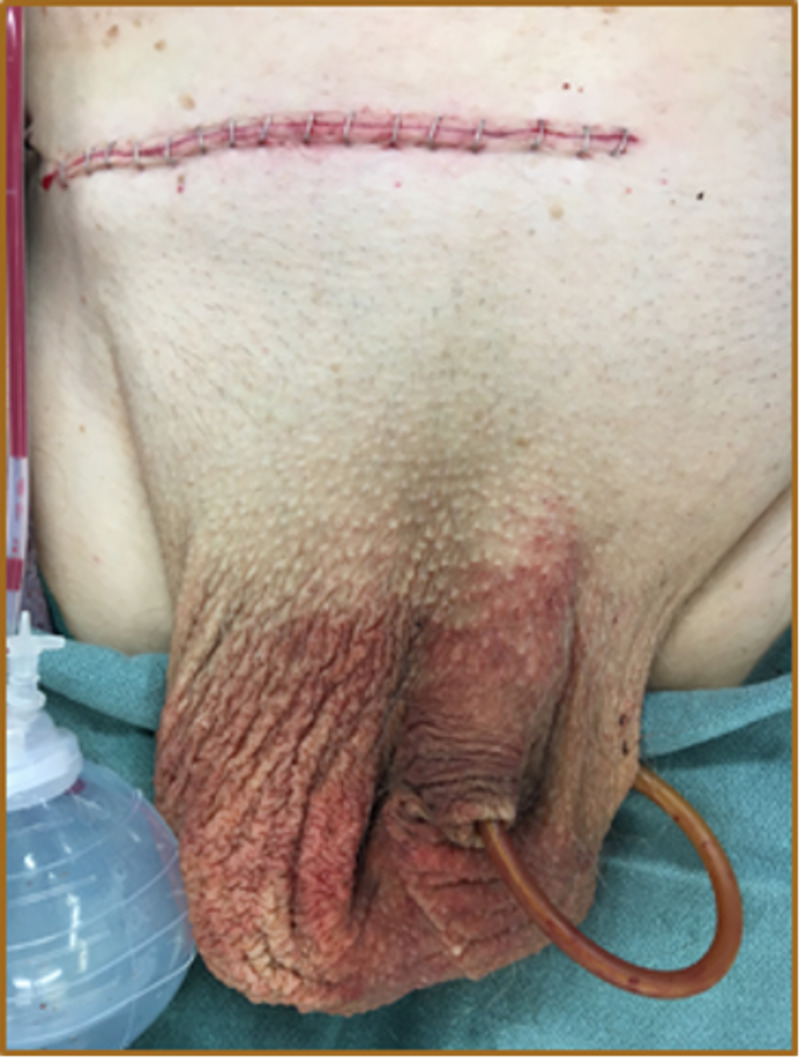
Finished repair with Jackson Pratt drain in the scrotum

Intra-operatively, the patient’s peak airway pressure was continuously monitored to evaluate for abdominal compartment syndrome. The patient’s pressures stayed within the normal limit. The hernia sac was then excised at the level of the internal ring and ligated using a non-absorbable suture. The resulting hernia defect measured approximately 5-6 centimeters. A tension-free repair using prolene mesh was performed (Figure 7). The external oblique aponeurosis was closed, taking care not to catch the ilioinguinal nerve. A suction drain was placed within the now huge empty right hemiscrotum. Scarpa's fascia was closed, and the skin was closed with a skin stapler (Figure 8).

**Figure 9:**
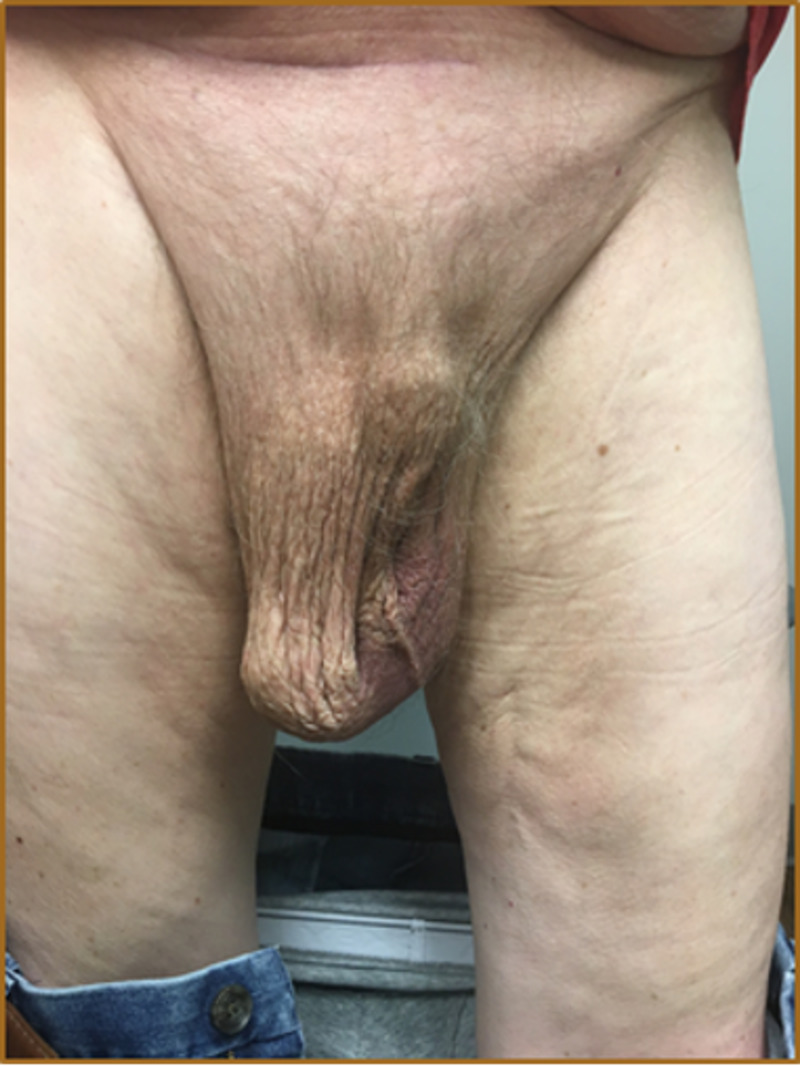
Five-month follow up

Postoperatively, the patient developed early satiety. Diet was slowly advanced with the scheduling of frequent, small meals. He was discharged home on post-op day 3 with a specific diet regimen for two weeks. At a five-month follow-up, the patient is doing well. The pain has resolved, the limitation in his ambulation has resolved, and he states a significantly improved quality of life (Figure 9).

## Discussion

In a retrospective analysis of 1647 inguinal hernia subjects by Mitura et al., only 1.1% of hernias were measured to be greater than 10 cm. In the same study, the average duration of hernia presentation in the subjects was found to be 25.4 months [4]. The inguinal hernia presented in this case surmounted both of these statistics by a large margin, depicting the severity of its progression.

Giant inguinal hernia repairs present with a unique set of risks and complications, especially the potential development of abdominal compartment syndrome.

Abdominal compartment syndrome (ACS) is a clinical manifestation of increased pressure within the abdominal cavity, usually beginning with intra-abdominal hypertension (IAH). IAH is defined as intra-abdominal pressure above 12 mmHg, and this may progress to ACS in critical patients when pressures reach above 20 mmHg [5, 6]. Peak inspiratory pressure may also be used as a surrogate measurement of ACS, as was done in this patient. ACS can lead to malfunction of major bodily systems, such as decreased lung compliance, decreased venous return, compression of the heart, and reduced perfusion to the abdominal organs [5].

The chronic and massive displacement of bowel contents from the abdominal cavity to the scrotum in an inguinal hernia may have physiological effects on the abdominal wall muscles that contribute to ACS upon reduction of the herniated contents. In rat model studies, chronic incisional hernia led to lateral abdominal wall shortening and internal oblique muscle atrophy and fibrosis. Chronic unloading of outward pressure on the abdominal wall muscles causes these changes that reduce elasticity and compliance [7]. These muscle studies have yet to be applied to humans, but we hypothesize that these same physiological changes occur in humans (as was seen by the gross atrophy of the external oblique aponeurosis in this patient) and contribute to the risk of ACS when reducing a giant inguinal hernia.

There are some techniques reported in the literature to minimize the intra-abdominal pressure during the surgical reduction of hernias in patients with LOD. Bueno-Lledo et al. described botulism-induced relaxation of abdominal wall muscles prior to surgery, as well as abdominal cavity expansion via catheter insufflation to accommodate the herniated volume [8]. Additional techniques reported include hemicolectomy and resection of small bowel in order to accommodate the reduction of herniated contents [9].

## Conclusions

Surgical management of giant inguinal hernia is challenging and poses higher risks when compared to normal-sized hernias, most notably an increase in intra-abdominal pressure and subsequent risk of ACS. Therefore, pre-operative planning and intra-operative monitoring during the reduction of hernia contents are critical for a successful repair.
